# LINC00665 sponges miR-641 to promote the progression of breast cancer by targeting the SNF2-related CREBBP activator protein (SRCAP)

**DOI:** 10.1080/21655979.2022.2031402

**Published:** 2022-02-12

**Authors:** Wen Cao, Xiaojing Liu, Weijia Su, Hao Liang, Huiru Tang, Weiliang Zhang, Shuhong Huang, Ningning Dang, Aiguo Qiao

**Affiliations:** a Health College, Yantai Nanshan University, Yantai, Shandong, China; b Department of Clinical Laboratory Medicine, Shandong University Qilu Hospital, Jinan, China; c Institute of Basic Medicine, Shandong Provincial Hospital Affiliated to Shandong First Medical University, Jinan, Shandong, China; d Department of Dermatology, Shandong Provincial Hospital Affiliated to Shandong First Medical University & Shandong Academy of Medical Sciences, Jinan, Shandong, China

**Keywords:** LINC00665, miR-641, SRCAP, ceRNA, 3ʹUTR

## Abstract

The regulatory network of competing endogenous RNAs (ceRNA) exists widely in tumors and affects the expression of cancer-related genes, thus playing an important role in the development and prognosis of human tumors. In this research, we explored the role and mechanism of LINC00665 as a ceRNA in breast cancer. We analyzed the expression and targets of LINC00665 in breast cancer using bioinformatics, and detected their effects on breast cancer cells by CCK8, transwell, colony formation and flow cytometry assays. From our results, LINC00665 knockdown suppressed the proliferation, migration and invasion and induced the apoptosis through inactivating the AKT/mTOR signaling pathway in MCF7 and MDA-MB-231 cells. LINC00665 had five potential downstream target miRNAs (miR-542-3p, miR-624-5p, miR-641, miR-425-5p, and miR-30-3p). In dual-luciferase report gene assay, the fluorescence activity of cells transfected with miR-641 mimics decreased, and the expression of miR-641 decreased significantly after knocking down LINC00665. miR-641 mimics significantly inhibited cell proliferation and invasion in MCF7 and MDA-MB-231 cells. We detected five potential direct targets of miR-641 using qPCR (SRCAP, SIKE1, NADK, KHDC4, and HSPG2). SRCAP expression decreased significantly in miR-641 overexpression cells and the binding of SRCAP’s 3ʹUTR and miR-641 was further confirmed by dual-luciferase report gene assay. SRCAP blocked the proliferation and invasion inhibition induced by miR-641 or si-LINC00665 in MCF7 and MDA-MB-231 cells. In conclusion, LINC00665 could promote the survival and metastasis of breast cancer cells through sponging miR-641 and targeting SRCAP. This research provided new potential targets for targeted therapy in human breast cancer.

## Background

In recent years, the incidence of breast cancer has increased year by year, accounting for the top cause of death in women with malignant tumors [1]. More than 1 million women are diagnosed with breast cancer each year worldwide, accounting for more than 20% of female cancers [2].

The hypothesis of competing endogenous RNAs (ceRNA) was first proposed in 2011 [3]. CeRNAs are non-coding RNAs that can bind to miRNA, thereby antagonizing the inhibitory effect of miRNA on coding genes and realizing the mutual communication between RNAs [4]. Many studies have shown that the regulatory networks of ceRNAs exist widely in human tumors and affects the expression of cancer-related genes, thus playing an important role in tumor development and prognosis [5].

Long-non-coding RNAs (lncRNAs) participate in the regulation of tumor cells as ceRNA [6,7]. Previous studies have shown that the highly expressed lncRNA DGCR5 can regulate the expression of multiple target genes, such as estrogen receptor 1, checkpoint kinase 1, fibroblast growth factor 2, sterile alpha motif domain containing 5 (Samd5), ephrin-A3, and forkhead box P3, thereby inhibiting the proliferation and lymphatic metastasis of triple-negative breast cancer (TNBC) cells [8]. LncRNA ROR plays an important role in the pathological mechanism of tumors, and acts as the ceRNA of YEB2 through competitively binding to miR-205 in breast cancer [9]. The high expression of ROR attenuates the inhibition of miR-205 on ZEB2 and promotes the expression of ZEB2, which can induce epithelial mesenchymal transformation (EMT) and metastasis of breast cancer [9]. Therefore, exploring ceRNAs can improve our understanding of tumor growth and metastasis mechanisms, and provide potential targets for bio-targeted therapy. Abnormal expression of Long Intergenic Non-Protein Coding RNA 665 (LINC00665) exists in a variety of tumors, including liver cancer [10,11], gastric cancer [12–14] and lung cancer [15,16]. In recent years, the role of LINC00665 in tumor has been gradually discovered, especially in breast cancer [17–22]. However, its mechanism in breast cancer as a ceRNA remains to be explored.

In the present research, we hypothesized that LINC00665 as a ceRNA involved in the progression of breast cancer. We aimed to investigated the specific role and mechanism of LINC00665 in tumor cells to deepening our understanding on the progression and metastasis of breast cancer.

## Material and methods

### Cell culture and transfection

Human normal mammary epithelial cell line MCF10A and breast cancer cell lines MCF7, MDA-MB-231, MDA-MB-453, and SK-BR-3 were all purchased from the American Type Culture Collection (ATCC) and cultured in Dulbecco’s Modified Eagle Medium (DMEM) with 10% fetal bovine serum. Transfection was performed using lipofectamine 3000 (Invitrogen, Life Technologies Corporation, Carlsbad, CA, USA) according to the manufacturer’s instructions. All plasmids used in this study were synthesized from RiboBio (Guangzhou, China).

### Quantitative real-time RT-PCR (qPCR)

Total RNA was extracted from each group after the transfection for 24 h using TRIzol reagent according to the manufacturer’s instructions. The reverse transcription and qPCR for miR-641 were performed using Taq Man MicroRNA kit and Taq Man Universal PCR Master-Mix (Applied Biosystems, Foster City, USA). The reverse transcription and qPCR for LINC00665, SNF2-related CREBBP activator protein (SRCAP), SIKE1, NADK, KHDC4, and HSPG2 were performed using HiFiScript cDNA Synthesis Kit and UltraSYBR Mixture (CwBio, Beijing, China) according to the manufacturer’s instructions by a qPCR thermal cycler (FTC-3000, Funglyn Biotech Inc., Toronto, Canada). The primers and sequences were provided in Table 1. Relative expression was analyzed using 2^−ΔΔCT^ method [23].

**Table 1.** Primers and sequences used in the present research

Name	Sequence (5’-3’)
si-LINC00665-sense strand	AAUAGCCCAAGACUGAGGACUCACA
si-LINC00665-antisense strand	UGUGAGUCCUCAGUCUUGGGCUAUU
LINC00665-Forward	AGCACCCCTAGTGTCAGT CA
LINC00665- Reverse	TGGTCTCTAGGGAGGCAGAA
SRCAP-Forward	CTCCACTGCTACCTCGTTTGGT
SRCAP-Reverse	GGAAAATCCGTTCCAGGCGTTC
SIKE1-Forward	AAAGCGGTGGATGCTGAACCAG
SIKE1-Reverse	CATCCACCTGAACTGCTTTCCTC
NADK-Forward	GTCCTTTGATGGACGGAAGAGAC
NADK-Reverse	GAGGCTCTCAAACCAGTCGCTC
KHDC4-Forward	AGAGGAGCTACCAGATGAACGG
KHDC4-Reverse	TGCCTGAGGAACTTGCTGGCTT
HSPG2-Forward	TCAGGCGAGTATGTGTGCCATG
HSPG2-Reverse	GATGAAGACTCGATCCTGACAGG
miR-641-Forward	GACATAGGATAGAGTCAC
miR-641-Reverse	GAACATGTCTGCGTATCTC
Actin-Forward	ATCAAGATCATTGCTCCTCCTG
Actin-Reverse	GTCATACTCCTGCTTGCTGAT
U6-Forward	CTCGCTTCGGCAGCACA
U6-Reverse	AACGCTTCACGAATTTGCGT

### Western blot and antibodies

The expression of target protein was detected using Western blot as described previously [24]. After transfection for 48 h, protein was extracted from each group by using a RIPA buffer with the complete protease inhibitor (Roche, USA) and measured by BCA method. The protein samples (20 μg) were separated using 10% SDS-PAGE gel, followed by transferred into a polyvinylidene fluoride (PVDF) membrane. Then, the membrane was incubated with primary antibodies at room temperature for 1 h and secondary antibody at room temperature for another 1 h. Antibodies of Bcl-2 (ab32124, 1:500), Bax (ab32503, 1:1000), Cleaved caspase3 (ab2302, 1:1000), AKT (ab8805, 1:1000), p-AKT (ab38449, 1:1000), mTOR (ab2732, 1:500), p-mTOR (ab109268, 1:500) were purchased from Abcam (Cambridge, UK). The chemiluminescence detection kit was used for signal development. The expression levels of target proteins were analyzed using ImageJ software.

### Cell counting kit (CCK8) and colony formation assays

CCK8 and colony formation assays were performed as described previously [24]. After transfection, 1 × 10^3^ cells were seeded into a 96-well plate. Cell viability was detected by CCK8 assay every 24 h after the transfection according to the manufacturer’s instructions. Optical density (OD) 450 value was measured on an iMark microplate reader (Bio-Rad, Munchen, Germany). The proliferation of MCF7 and MDA-MB-231 cells was further detected by colony formation assay. After the transfection, cells were transferred into 6 cm plates at a density of 500 cells/plate. Cells were incubated at 37°C for 14–21 days. Then, cells were stained with giemsa for 15 min. Colony was counted by two researchers in five random fields.

### Transwell assay

Transwell assay was performed to detect the migration and invasion of MCF7 and MDA-MB-231 cells as described previously [24]. The chilled Matrigel was diluted with serum-free DMEM at 1:6. 100 μl of Matrigel was applied to the transwell chamber and incubate for 4 h. After transfection, about 1 × 10^4^ cells, which were suspended in serum-free medium were seeded into the upper chamber of the transwell. After incubated for 48 h, cells were stained with 0.1% crystal violet for 5 min. The invaded cells were imaged and counted under a microscope at a magnification of × 200. For migration assay, the transwell was not processed with Matrigel.

### Flow cytometry analysis

After transfection for 48 h, 5 μl AnnexinV‐FITC was added into 100 μl cell suspension (3–5 × 10^5^ cells/ml), followed by 10 μl of PI. Cells were analyzed using a flow cytometer (BD FACSC anto II, BD Biosciences, San Jose, CA, USA). Cell apoptosis was calculated using the BD FACSDiva software [24].

### Dual-luciferase report gene analysis

The 3’-untranslated region (UTR) of SRCAP or LINC00665 containing the wild or mutant type of binding sites were cloned into pGL3 plasmid. The luciferase plasmids were transfected into HEK 293 T cells. After the transfection, the luciferase expression was determined by a Dual-Glo Luciferase Assay System (Promega, USA) and normalized to wild-type group [25].

### Statistical analysis

All data were presented as mean ± s.d. Each experiment was performed in triplicate. Student’s t test and one-way ANOVA were applied for the comparison between groups. Post hoc was performed using Bonferroni method in the multiple comparison with ANOVA. The correlation between groups was evaluated using Pearson Correlation Coefficient. Data analysis and graph generation were all performed using GraphPad Prism 8. A value of P < 0.05 was considered statistically significant.

## Results

In the present research, we investigated the effect and molecular mechanism of LINC00665 as a ceRNA in breast cancer cells, and confirmed that LINC00665/miR-641/SRCAP axis regulates tumor cell survival and metastasis through EMT and AKT pathway. The expression correlation of LINC00665/miR-641/SRCAP was verified in clinical samples of breast cancer patients.

### Knockdown of LINC00665 suppressed the proliferation in breast cancer cells

**Figure 1.**
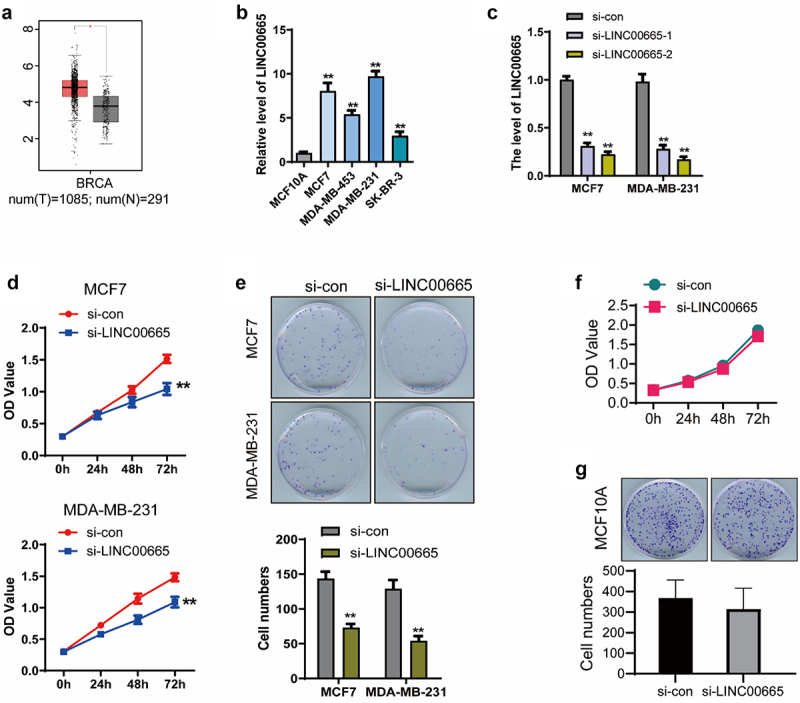
Knockdown of LINC00665 suppressed cell proliferation in breast cancer cells.

Firstly, we analyzed the expression of LINC00665 in breast invasive carcinoma (BRCA) using the online dataset GEPIA (http://gepia.cancer-pku.cn/index.html) [26]. As shown in Figure 1a, the level of LINC00665 in BRCA samples (n = 1085) was significantly higher than that in breast normal samples (n = 291) (P < 0.05), indicating that LINC00665 involved in the progression of human breast cancer. Then, the level of LINC00665 was detected using qPCR in four breast cancer cell lines with the human normal mammary epithelial cell line MCF10A as a control. As shown in Figure 1b, the levels of LINC00665 in MCF7, MDA-MB-231, MDA-MB-453 and SK-BR-3 were markedly up-regulated compared with that in MCF10A cells (P < 0.01).

(A) The expression of LINC00665 in BRCA was obtained from the online dataset GEPIA (http://gepia.cancer-pku.cn/index.html). |Log2FC| Cutoff = 1. log_2_(TPM + 1) was used for log-scale. (B) Relative level of LINC00665 was detected using qPCR in four breast cancer cell lines with the human normal mammary epithelial cell-line MCF10A as a control. (C) LINC00665-siRNA (si-LINC00665) was transfected into breast cancer cell lines MCF7 and MDA-MB-231 cells with the negative siRNA as a control (si-con). The level of LINC00665 was detected by qPCR. (D) Cell activity was detected by CCK8 assay. (E) The proliferation of si-LINC00665 and si-con cells was further confirmed by clonogenic assay. The plates were culture for 14 days after the transfection and the numbers of cell clones in five random fields were counted by two independent researchers. (F) LINC00665-siRNA was transfected into MCF10A cells with the negative siRNA as a control, and cell viability was detected using CCK8 assay. (G) The proliferation of MCF10A was detected using clonogenic assay. *P < 0.05; **P < 0.01.

We synthesized LINC00665-specific siRNA and transfected it into the two cell lines with the highest expression of LINC00665. LINC00665 expression was significantly inhibited by the transfection of LINC00665-siRNA in MCF7 and MDA-MB-231 cells (Figure 1c, p < 0.01). CCK8 and clonogenic assays were performed to detect cell proliferation. As shown in Figure 1d, the OD450 decreased after the transfection of LINC00665-siRNA (MCF7, 1.04 ± 0.09; MDA-MB-231, 1.09 ± 0.08) for 72 h compared with the control cells (MCF7, 1.52 ± 0.06; MDA-MB-231, 1.48 ± 0.06) (P < 0.01). Colony numbers of si-LINC00665 group (MCF7, 73 ± 10; MDA-MB-231, 54 ± 12) was also declined compared with the si-con group (MCF7, 144 ± 17; MDA-MB-231, 129 ± 22) (Figure 1e, p < 0.01).

Then, we performed these experiments in MCF10A cell line to determine whether the effect of LINC00665 on cell proliferation is restricted to tumor cells only. As shown in figure 1f, the OD450 had no significant decrease in LINC00665 knockdown MCF10A cells compared with the control group (P > 0.05). Consistently, there was no significant difference in the colony numbers between these two groups (Figure 1g, p > 0.05). These results proved that knockdown of LINC00665 specifically inhibited the proliferation of human breast cancer cells.

### Knockdown of LINC00665 inhibited the migration and invasion in breast cancer cells

**Figure 2.**
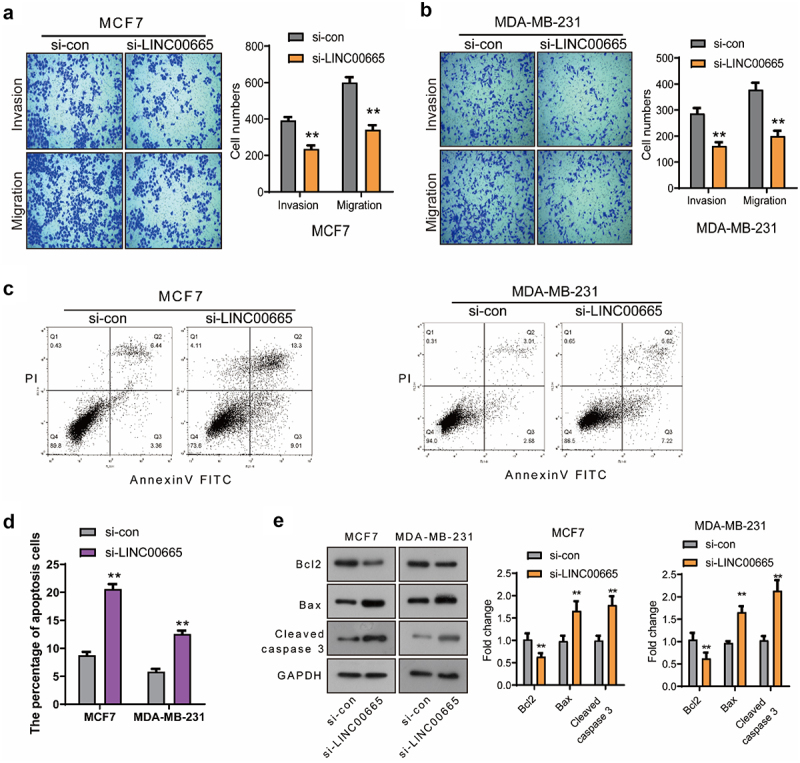
Knockdown of LINC00665 inhibited the migration and invasion and induced the apoptosis in breast cancer cells.

Next, the effect of LINC00665 on the migration and invasion of MCF7 and MDA-MB-231 cells was detected using transwell assay. As shown in Figure 2a and 2B, the migrated cell number declined markedly after the transfection of LINC00665-siRNA (MCF7, 341 ± 44; MDA-MB-231, 200 ± 36) compared with the control siRNA (MCF7, 600 ± 49; MDA-MB-231, 378 ± 46) (P < 0.01). Moreover, the number of invasive cells in si-LINC00665 group (MCF7, 236 ± 33; MDA-MB-231, 162 ± 25) was also decreased compared with the si-con group (MCF7, 392 ± 32; MDA-MB-231, 286 ± 37) (P < 0.01). These data proved that LINC00665-siRNA could inhibit the migration and invasion in breast cancer cells.

Transwell assay was performed to detect the migration and invasion in MCF7 (A) and MDA-MB-231 (B) cells. (C, D) Flow cytometry was performed to analysis the apoptosis of si-LINC00665 and si-con cells. (E) The expression of apoptosis-related proteins Bcl-2, Bax, and Cleaved caspase 3 was detected using Western blot. **P < 0.01.

### Knockdown of LINC00665 induced the apoptosis in breast cancer cells

We detected the apoptosis of si-LINC00665 and si-con cells using flow cytometry analysis. As shown in Figure 2c and 2D, the percentage of apoptotic cells increased after the transfection of LINC00665-siRNA (MCF7, 20.57 ± 1.57%; MDA-MB-231, 12.55 ± 1.09%) compared with that of the control cells (MCF7, 8.74 ± 1.06%; MDA-MB-231, 5.80 ± 0.89%) (P < 0.01). To study the mechanism by which si-LINC00665 induced cell apoptosis, the expression levels of apoptosis-related proteins Bcl-2, Bax and Cleaved caspase 3 were detected using Western blot. The transfection of LINC00665-siRNA down-regulated the level of Bcl-2 and up-regulated the levels of Bax and Cleaved caspase 3 (Figure 2e, p < 0.01). These results indicated that LINC00665-siRNA could induce the apoptosis through regulating the apoptosis factors in MCF7 and MDA-MB-231 cells.

### Knockdown of LINC00665 inactivated the AKT/mTOR signaling pathway in breast cancer cells

**Figure 3.**
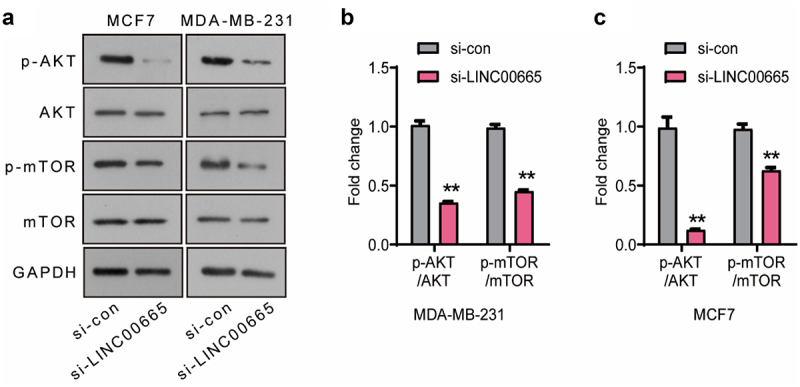
Knockdown of LINC00665 inactivated the AKT/mTOR signaling pathway in breast cancer cells.

The AKT/mTOR signaling pathway is abnormally activated in a variety of human tumors, and plays an important role in tumor cell proliferation, survival, apoptosis, angiogenesis, metastasis, and resistance to radiotherapy and chemotherapy. In the present research, the expression levels of p-AKT, AKT, p-mTOR and mTOR were analyzed using Western blot (Figure 3a). As shown in Figure 3b and 3C, the levels of p-AKT/AKT and p-mTOR/mTOR of si-LINC00665 group declined significantly compared with si-con group in MCF7 and MDA-MB-231 cells (P < 0.01), suggesting that LINC00665-siRNA inhibited the survival of breast cancer cells through inactivating the AKT/mTOR signaling pathway.

(A) The expressions of p-AKT, AKT, p-mTOR, and mTOR were analyzed using Western blot. The levels of p-AKT/AKT and p-mTOR/mTOR in MCF7 (B) and MDA-MB-231 (C) cells were analyzed using ImageJ. **P < 0.01.

### LINC00665 sponged miR-641 in breast cancer cells

**Figure 4.**
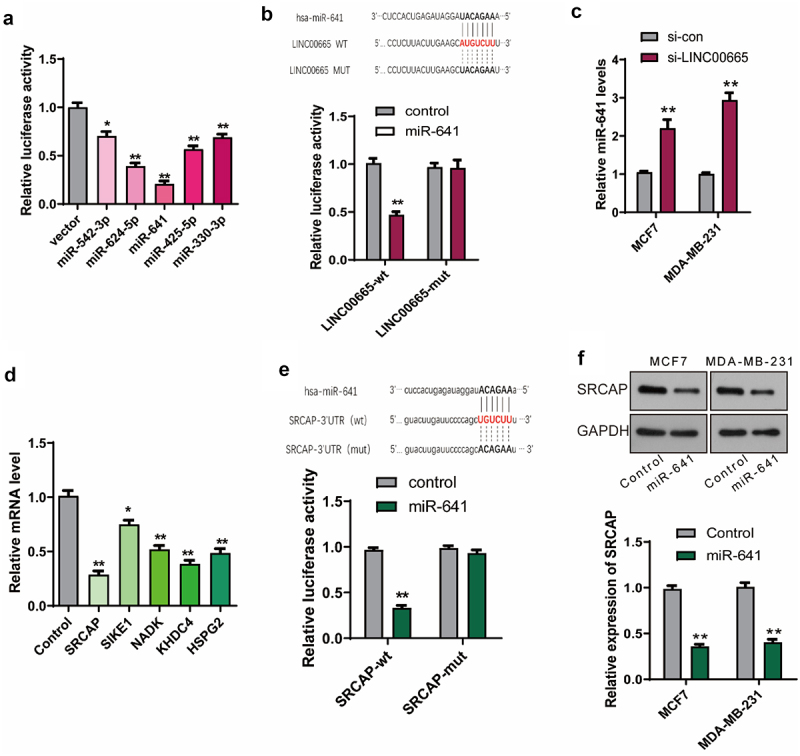
LINC00665 promoted the expression of SRCAP through sponging miR-641 in breast cancer cells.

Then, we explored the molecular mechanism by which LINC00665 promoted breast cancer. The potential miRNAs which could bind to LINC00665 were analyzed using the online software StarBase (http://starbase.sysu.edu.cn/starbase2/index.php) [27]. As shown in Figure 4a, miR-542-3p, miR-624-5p, miR-641, miR-425-5p, and miR-30-3p were selected for further validation in combination with LINC00665. LINC00665 sequence was constructed into a pGL3 plasmid (LINC00665-wt), and the combination of LINC00665 with miRNAs was analyzed using dual-luciferase report gene assay. The relative luciferase activity decreased significantly after the transfection of miR-542-3p, miR-624-5p, miR-641, miR-425-5p, or miR-30-3p, and decreased most significantly after miR-641 transfection. Therefore, we further constructed a LINC00665-mut plasmid containing a sequence of LINC00665 with mutation-binding site (UACAGAA). As shown in Figure 4b, there was no significant change in luciferase activity after transfection with LINC00665-mut plasmid compared with the control group. These results proved that LINC00665 bound to miR-641 via the predicted sites (AUGUCUU). The level of miR-641 was further detected using qPCR in si-LINC00665 and si-con group cells. As shown in Figure 4c, the miR-641 level increased after the transfection of LINC00665-siRNA both in MCF7 and MDA-MB-231 cells (P < 0.01).

(A) MiRNAs, which could bind to LINC00665, were analyzed using the online software StarBase. The sequence of LINC00665 was constructed into pGL3 plasmid (LINC00665-wt), and then dual-luciferase report gene analysis was performed to detect the combination of LINC00665 and miRNAs (miR-542-3p, miR-624-5p, miR-641, miR-425-5p, and miR-30-3p). (B) The sequence of LINC00665 with mutation-binding site (UACAGAA) was constructed into pGL3 plasmid to generate LINC00665-mut plasmid (upper panel). The combination of LINC00665 and miR-641 was further confirmed using dual-luciferase report gene analysis (bottom panel). (C) The miR-641 level was detected using qPCR in si-LINC00665 and si-con group. (D) Potential targets of miR-641 were analyzed using the online software StarBase. qPCR was performed to detect the levels of miR-641ʹs potential targets (SRCAP, SIKE1, NADK, KHDC4, and HSPG2) after the transfection of miR-641 mimics. (E) The sequence of SRCAP-3ʹUTR with wild-type (UGUCUU) or mutation-binding site (ACAGAA) was constructed into pGL3 plasmid to generate SRCAP-wt or SRCAP-mut plasmid, respectively (upper panel). The combination of SRCAP and miR-641 was confirmed using dual-luciferase report gene analysis (bottom panel). (F) The SRCAP level was detected using qPCR after the transfection of miR-641 mimics with the untreated MCF7 and MDA-MB-231 cells as control groups. *P < 0.05; **P < 0.01.

### MiR-641 inhibited the expression of SRCAP via binding to its 3ʹUTR

The potential targets of miR-641 were analyzed using the online software StarBase [27] and verified using qPCR. As shown in Figure 4d, miR-641 mimics decreased the levels of SRCAP, SIKE1, NADK, KHDC4, and HSPG2, especially SRCAP compared with the control cells. Then, the combination of miR-641 with SRCAP was further confirmed using dual-luciferase report gene assay. The sequence of SRCAP-3ʹUTR with wild-type (UGUCUU) or mutation-binding site (ACAGAA) was constructed into pGL3 plasmid to generate SRCAP-wt or SRCAP-mut plasmid, respectively. As shown in Figure 4e, SRCAP-wt but not SRCAP-mut significantly inhibited luciferase activity (P < 0.01), indicating that miR-641 could bind to the 3ʹUTR of SRCAP (UGUCUU). The SRCAP level was further detected using qPCR after the transfection of miR-641 mimics with the untreated MCF7 and MDA-MB-231 cells as control groups. As shown in figure 4f, SRCAP was down-regulated by the transfection of miR-641 mimics (P < 0.01).

### MiR-641 inhibited the proliferation and invasion in breast cancer cells

**Figure 5.**
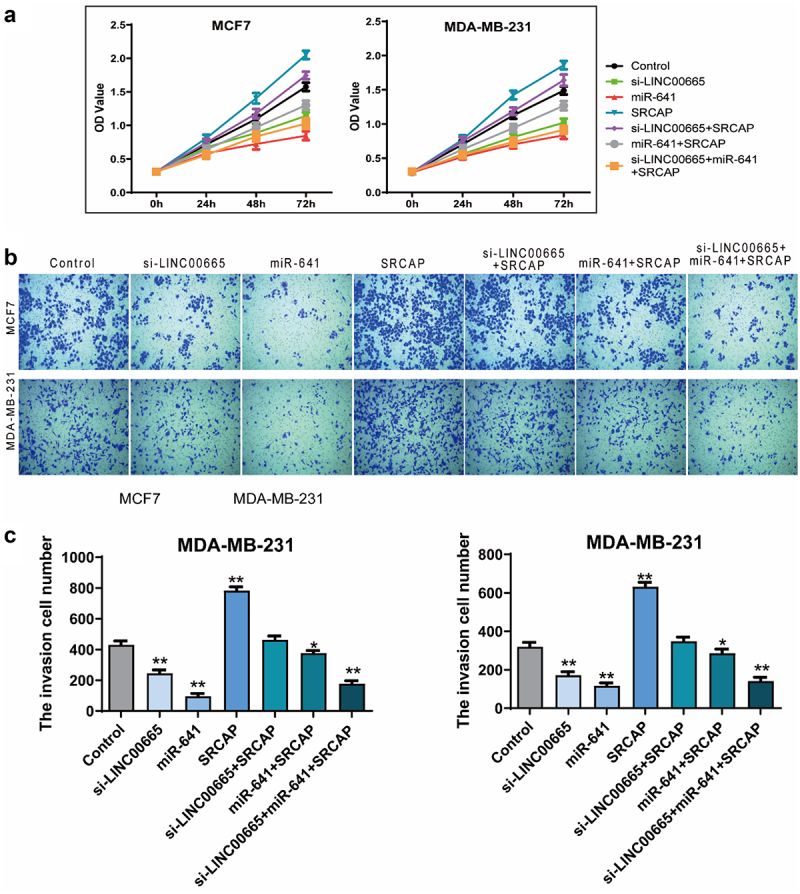
Knockdown of LINC00665 inhibited the proliferation and invasion through down-regulating SRCAP by sponging miR-641.

We detected the effect of miR-641 mimics on the proliferation and invasion of MCF7 and MDA-MB-231 cells using CCK8 and transwell assay. As shown in Figure 5a, the OD450 markedly declined after the transfection of miR-641 mimics for 72 h (P < 0.01). Moreover, the invasive cell number also declined after the transfection of miR-641 mimics (MCF7, 96 ± 30; MDA-MB-231, 117 ± 26) compared with the untreated cells (MCF7, 431 ± 44; MDA-MB-231, 319 ± 72) (Figure 5b-C, P < 0.01).

(A) CCK8 was performed to detect the proliferation in MCF7 and MDA-MB-231 cells. (B and C) Transwell assay was used to detect the invasion in MCF7 and MDA-MB-231 cells.

### SRCAP blocked the proliferation inhibition induced by miR-641 or si-LINC00665

Next, we overexpressed SRCAP in the LINC00665 knockdown cells, as well as the miR-641 overexpressed cells to explore whether SRCAP was the downstream target of LINC00665/miR-641. From the results of CCK8, SRCAP overexpression could significantly increase OD450 value and block the decrease of OD450 value caused by miR-641 mimics or LINC00665 knockdown (Figure 5a, p < 0.01). Moreover, compared with the control group (MCF7, 431 ± 44; MDA-MB-231, 319 ± 72), SRCAP overexpression (MCF7, 783 ± 41; MDA-MB-231, 631 ± 43) significantly increased the number of invasive cells (P < 0.01); compared with the miR-641 group (MCF7, 96 ± 30; MDA-MB-231, 117 ± 26), SRCAP overexpression (MCF7, 376 ± 30; MDA-MB-231, 286 ± 38) increased invasive cell number (P < 0.05); compared with the si-LINC00665 group (MCF7, 245 ± 39; MDA-MB-231, 171 ± 32), SRCAP (MCF7, 462 ± 45; MDA-MB-231, 348 ± 39) also increased invasive cell number with statistically significant (P < 0.01) (Figure 5b and c). These data indicated that si-LINC00665/miR-641 involved in the regulation of biological behavior in breast cancer cells via targeting SRCAP.

### Knockdown of LINC00665 reversed the EMT process

**Figure 6.**
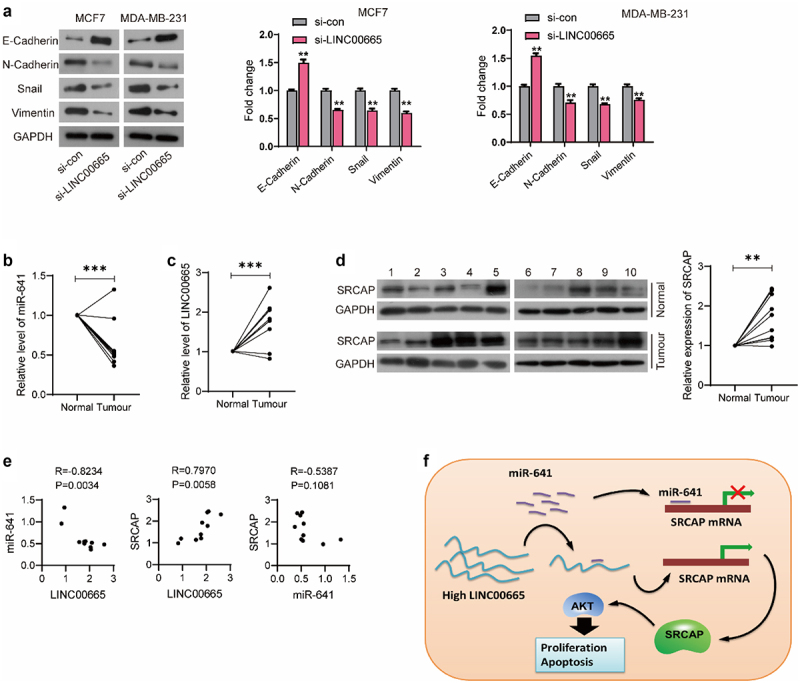
LINC00665 was up-regulated in breast cancer patients.

EMT is a phenomenon of epithelial cells transforming into interstitial cells, and is closely related to the occurrence, development, invasion and metastasis of malignant tumors. Our results showed that LINC00665-siRNA could up-regulate the level of epithelial marker E-Cadherin (P < 0.01), and down-regulate the levels of interstitial markers N-Cadherin, Snail, and Vimentin both in MCF7 and MDA-MB-231 cells (P < 0.01) (Figure 6a).

(A) The expression of EMT makers were detected using Western blot. (B) The expression of LINC00665 in clinical samples of 10 breast cancer patients was detected using qPCR. (C) The level of miR-641 was also detected using qPCR. (D) SRCAP expression was determined using Western blot. (E) The correlation between the expression of LINC00665, miR-641, and SRCAP in tumor tissues was evaluated using Pearson Correlation Coefficient. (F) Schematic diagram of LINC00665/miR-641/SRCAP axis promoting tumor cell proliferation. **P < 0.01; ***P < 0.001.

### The expression of LINC00665, miR-641, and SRCAP in breast cancer tissues

To further verify our hypothesis, we detected the expression levels of LINC00665, miR-641, and SRCAP in clinical samples from 10 breast cancer patients. As shown in Figure 6b, miR-641 level of tumor tissues was significantly lower than that of the normal tissues (P < 0.001). LINC00665 was highly expressed in the breast cancer samples (Figure 6c, p < 0.001). From the result of Western blot, SRCAP was up-regulated in breast cancer tissues compared with the normal tissues (Figure 6d, p < 0.01). Pearson correlation analysis showed that LINC00665 was negatively correlated with miR-641 level (P < 0.01) and positively correlated with the expression of SRCAP with statistical significance (P < 0.01); while SRCAP was negatively correlated with miR-641 without statistical significance (P > 0.05) (Figure 6e).

Our results proves that miR-641 inhibits the translation of SRCAP by binding to its 3ʹUTR region, while high-level LINC00665 promotes SRCAP expression through the sponge adsorption of miR-641, thus promoting the proliferation and metastasis of tumor cells (figure 6f).

## Discussion

In this study, we explored the role and mechanism of LINC00665 in breast cancer. First, we investigated LINC00665ʹs effect on the biological behavior of breast cancer cells by knocking down LINC00665 in MCF7 and MDA-MB-231 cells. Our results proved that knockdown of LINC00665 inhibited the proliferation, migration and invasion and induced the apoptosis, indicating a carcinogenesis role of LINC00665 in human breast cancer. Then, we found that knocking down LINC00665 can reverse EMT. Zhou et al. also confirmed that LINC00665 can activate the EMT pathway to promote breast cancer metastasis [19]. In recent years, the role of LINC00665 in tumor has been found gradually. LINC00665 is highly expressed in hepatocellular carcinoma and associated to the prognosis, and may participate in cell cycle regulation according to the results of bioinformatics and clinical sample analysis [10]. LINC00665 was identified as a biomarker of high risk oral premalignant lesions on the basis of lncRNA expression profiling analysis, and the target genes of LINC00665 were markedly enriched in the ubiquitin-protein ligase activity, ubiquitin-dependent protein catabolic process, and neurotrophin signaling [28]. In non-small-cell lung cancer (NSCLC), LINC00665 knockdown inhibits the proliferation and restores gefitinib sensitivity via the regulation of EZH2 and PI3K/AKT signaling pathway [29]. LINC00665 plays an important role in breast cancer. Analysis of samples from 102 breast cancer patients showed that LINC00665 is an independent predictor of pathological complete response and can predict treatment efficacy in neoadjuvant chemotherapy [22]. LINC00665 is highly expressed in breast cancer tissues and promotes tumor cell survival [21]. In addition, LINC00665 can also encode micropeptide CIP2A-BP, which is low expressed in tumor samples and reduces lung metastasis of triple-negative breast cancer in vivo [17]. This confirms the diverse regulatory network of LINC00665 in breast cancer.

Many studies have shown that LINC00665 is involved in the progression of breast cancer as a ceRNA [18,20]. In this study, bioinformatics analysis showed that LINC00665 operated as a ceRNA to sponge miR-641 and promote the expression of SRCAP in MCF7 and MDA-MB-231 cells, and their combination was verified by luciferase experiment. Recent studies have shown that miR-641 expression is reduced in lung cancer tissues, and miR-641 overexpression significantly inhibits the cell survival by targeting MDM2 in lung cancer cells [30,31]. MiR-641 is down-regulated in glioblastoma, and involves in the development of glioblastoma by regulating the PI3K/AKT signaling pathway [32]. However, Yanling et al. reports that knockdown of miR-641 can inhibit the proliferation, migration, and invasion of cervical cancer cells [33]. The above studies indicate that miR-641 plays different roles in different type of human tumors. Meanwhile, the role of miR-641 in breast cancer has not been reported. In this study, we found that miR-641 inhibited the proliferation and invasion in breast cancer cells. In addition, miR-641 also inhibited the expression of SRCAP by directly binding to the 3ʹUTR of SRCAP.

SRCAP is an ATP dependent chromatin remodeling enzyme of the INO80 family [34]. SRCAP has a total length of more than 3000 amino acids, contains an ATPase structure, an N-terminal helicase-SANT-associated (HSA) domain, a C-terminal AT-hook DNA binding motif, and a CBP binding domain in the middle [35]. The absence of the C-terminal AT-hook structure in SRCAP is the main cause of Floating-Harbor syndrome (FHS) [36]. In human tumors, SRCAP has only been reported in prostate cancer [37]. As a co-activator for the androgen receptor (AR), SRCAP promotes the proliferation and mediates the expression of prostate-specific antigen in prostate cancer [37]. In the present study, we proved that SRCAP promoted the proliferation and invasion, and blocked the inhibition of cell proliferation and invasion induced by miR-641 mimics or LINC00665 knockdown, suggesting that SRCAP was the direct target of LINC00665/miR-641 axis. In addition, SRCAP operated as a transcriptional activator in the cAMP response element-binding protein, steroid receptor and Notch-mediated transcription [38]. Seo J, et al. investigates the target genes of SRCAP in T lymphocyte by using transcription factor ChIP-seq [39]. However, it remains unclear about the targets of SRCAP in tumors. Therefore, we predicted that SRCAP involved in the regulation of tumor cell proliferation and metastasis through the regulation of specific-genes’ transcription, which need further study.

Finally, we confirmed in clinical samples that LINC00665 and SRCAP are highly expressed in tumor tissues, while miR-641 is low in tumor tissues. Consistent with the predicted results, LINC00665 was positively correlated with the expression of SRCAP, and negatively correlated with the expression of miR-641 in tumors. However, SRCAP was negatively correlated with the miR-641 without statistically significant, indicating a complex regulatory network of SRCAP expression in tumors. These results suggest that LINC00665, miR-641 and SRCAP may be potential targets for breast cancer diagnosis and biological treatment.

## Conclusion

We demonstrate that LINC00665 is overexpressed in breast cancer, and up regulated the expression of SRCAP through sponging miR-641 to finally promote the survival and metastasis of breast cancer cells. This research expands our understanding of the mechanism of LINC00665 role and provides new potential targets for bio-targeted therapy in human breast cancer.

## Data Availability

The data supporting the conclusions of this paper are included within the manuscript.

## Highlights

1. LINC00665 promotes the survival and metastasis of breast cancer cells.

2. LINC00665 sponges miR-641 to promote SRCAP expression.

3. LINC00665 downregulated in breast cancer tissues.
